# Intestinal Puncture in Tympanites

**Published:** 1869-08

**Authors:** 


Intestinal Puncture in Tympanites.—Under the advice of Dr. Fonssagrives, intestinal puncture, as a last resource, has been several times practised at Toulouse, on two patients suffering with tympanites. In the first case, the abdomen formed an immense mass; the patient was perfectly cyanosed and suffocating. An exploring trocar was inserted into the most distended part of the umbilical region. The gas escaped so violently as to extinguish a candle. The distension returning the next day, two fresh punctures were made in different places, and gave so much relief that the life of the patient was prolonged four days. In another case six punctures were successively made until the gases were naturally evacuated, and the patient cured.—*Western Journal of Medicine.*

